# A systematic review and coordinate-based meta-analysis of fMRI studies on acupuncture at LR 3

**DOI:** 10.3389/fnins.2024.1341567

**Published:** 2024-01-29

**Authors:** Yawen Rao, Limin Ge, Jiaxin Wu

**Affiliations:** The First Clinical Medical College, Guangzhou University of Chinese Medicine, Guangzhou, China

**Keywords:** acupuncture, fMRI, LR3 (taichong), brain activation, systematic review

## Abstract

**Objectives:**

The acupoint LR3 (Taichong) is frequently utilized in clinical acupuncture. However, its underlying neural mechanisms remain not fully elucidated, with speculations suggesting its close association with specific brain activity patterns.

**Methods:**

A comprehensive literature search was undertaken across several online databases, such as PubMed, Web of Science, Embase, Cochrane Library, CNKI (China National Knowledge Infrastructure), Wanfang Database, VIP Database, and the Chinese Biomedical Database. Two independent researchers handled the study selection, quality assessment, and data extraction processes. Using the seed-based d-mapping meta-analysis approach, we evaluated the brain regions activated by LR3 acupuncture in healthy subjects. Subsequent subgroup analysis was stratified by fMRI types, and regression analyses were performed considering the duration of acupuncture, depth of needle insertion, and needle diameter. The identified active brain regions were then intricately projected onto large-scale functional networks.

**Results:**

A total of 10 studies met the criteria for inclusion, encompassing 319 healthy right-handed participants. The meta-analysis indicates that acupuncture at the LR3 activates regions such as the right postcentral gyrus, left thalamus, left middle frontal gyrus, and right superior frontal gyrus. Additionally, meta-regression analysis highlights that increased acupuncture duration correlates with progressively intensified activation of the right superior frontal gyrus. Subgroup analysis posits that variations in the type of fMRI employed might account for heterogeneity in the pooled results. Concurrently, functional network analysis identifies the primary activated regions as aligning with the Basal ganglia network, Auditory network, Left executive control network, Posterior salience network, Right executive control network, and Sensorimotor networks.

**Conclusion:**

Acupuncture at the LR3 in healthy subjects selectively activates brain regions linked to pain perception, emotional processing, and linguistic functions. Extending the needle retention duration intensifies the activation of the right superior frontal gyrus. These findings enrich our comprehension of the neurobiological underpinnings of acupuncture’s role in pain mitigation and emotional regulation.

## 1 Introduction

Acupuncture is a key component of Traditional Chinese Medicine (TCM) with over 2,000 years of history. In recent decades, acupuncture has been increasingly embraced in Western alternative and complementary medicine (Cai et al., 2018). While acupuncture therapy has been employed to alleviate pain (He et al., 2020; Sun et al., 2021) and manage a range of conditions, including Parkinson’s Disease (Chae et al., 2009; Jang et al., 2020), cardiovascular and cerebrovascular disorders (Yang et al., 2018, 2021; Zhang et al., 2021), depression (Yang et al., 2022; Yin et al., 2022), addiction (Krause et al., 2020; Lee et al., 2021), and more (Tu et al., 2021; Yin et al., 2023), its efficacy and specificity of acupoint remain debated.

Acupuncture entails the insertion of metallic needles into specific acupuncture points strategically located on the human body, primarily within the skin and muscles, to achieve varied therapeutic effects. Animal experiments have revealed that acupuncture not only induces local effects (Chen et al., 2023), modulating the neuro-endocrine-immune microenvironment surrounding the acupuncture points but also activates the skin-brain axis, thereby achieving comprehensive local and systemic neuro-endocrine-immune regulation (Zhang X. N. et al., 2022). Furthermore, the efficacy of acupuncture closely correlates with the integration of the central nervous system (Han, 2011). In recent years, neuroimaging technologies such as functional magnetic resonance imaging (fMRI) (Li et al., 2017; Wang et al., 2020), resting-state functional near-infrared spectroscopy (Wong et al., 2021; Liu Y. et al., 2022), positron emission tomography (Lu Y. J. et al., 2017; Cai et al., 2019), and electroencephalography (Yu et al., 2018; Peng et al., 2019) have been extensively employed to investigate the brain’s response to acupuncture stimulation. Functional magnetic resonance imaging (fMRI) is a non-invasive, high-resolution magnetic resonance imaging technique based on Blood Oxygen Level-dependent (BOLD) signals, facilitating the quantification of acupuncture’s impact on the brain.

Taichong (LR3), a pivotal acupuncture point along the Liver Meridian of Foot Jueyin in traditional Chinese medicine, is believed to harbor functions such as emotion regulation, pain alleviation, and blood pressure reduction. Relevant fMRI studies suggest that the activation of LR3 correlates with brain regions including the cingulate gyrus, precuneus, posterior hippocampus, posterior cingulate gyrus, superior frontal gyrus, and posterior cingulate gyrus (Claunch et al., 2012; Qiu et al., 2012; Zhang et al., 2015). However, findings regarding specific brain regions and the direction of activation changes within these regions often exhibit inconsistency, possibly attributed to limited sample sizes and methodological variations (Radua and Mataix-Cols, 2012).Meta-analysis in the field of neuroimaging provides an effective approach to ascertain the consistency among datasets and enhances statistical power (Marwood et al., 2018).

Therefore, in this study, we employed Seed-based d Mapping (SDM) technology and functional network mapping methods to explore the impact of acupuncture at the LR3 single acupoint on brain function. Concurrently, through subgroup analysis and meta-regression analysis, we delved deeper into the brain activation patterns during acupuncture at the LR3 acupoint, aiming to elucidate potential neurobiological mechanisms of acupuncture at LR3.

## 2 Method

This study presents a systematic review and meta-analysis of the neural activities of LR3 acupuncture stimulation in healthy subjects. The research strictly adhered to Preferred Reporting Items for Systematic Reviews and Meta-Analyses (PRISMA) and for Acupuncture (PRISMA-A) guidelines. The protocol was duly registered in the Prospective Register of Systematic Reviews (CRD42022380728).

### 2.1 Search strategy

A systematic search of multiple electronic databases was conducted to identify pertinent studies. Four English databases—PubMed, Web of Science, Embase, and Cochrane Libraries—and four Chinese databases—China National Knowledge Infrastructure, Wanfang, WeiPu, and China Biology Medicine—were included in the search. No specific start date constrained the search, which was updated until June 2023.The following terms and their derivatives were used in each database: (“LR 3” or “LR3” or “taichong”) and (“fMRI” or “functional MRI” or “functional magnetic resonance imaging”).

### 2.2 Selection criteria

The criteria for study inclusion entailed: (1) research on single “LR3” acupoint stimulation, (2) involvement of healthy adults with detailed demographic data, (3) exploration of acupuncture’s immediate effect on brain activity, including both resting-state and task-state fMRI, (4) application of whole-brain analysis to fMRI data, and (5) provision of peak stimulus coordinates in standardized anatomical spaces such as Talairach or Montreal Neurological Institute (MNI), supplemented by the corresponding cluster size and statistical metrics (e.g., voxel-wise value of ps, z values, or t scores). Studies were excluded if they were (1) duplicative, (2) utilized region of interest (ROI) analysis for fMRI data, or (3) classified as reviews, meta-analyses, case studies, or animal research. We employed a modified quality assessment scale (Iwabuchi et al., 2015), based on Stroup’s MOOSE statement (Stroup et al., 2000), with a maximum score of 20—scores of 15 or above denoted high quality. Authors RYW and GLM independently executed the study search and screening according to these criteria. The authors individually evaluated each publication.

### 2.3 Data extraction

Two authors (RYW and GLM) independently extracted data from the selected studies utilizing a predetermined data extraction form. This form captured: first authors’ names, publication years, participant demographics, needle duration durations, needle diameters, study designs (either resting-state or task-state fMRI), MRI acquisition methodologies, neuroimaging processing software, coordinate systems, and peak coordinates in the brain regions accompanied by their t-values.

### 2.4 Statistical analysis

In this study, a voxel-based meta-analysis was performed using SDMv5.1 software (Seed-based d Mapping, http://www.sdmproject.com/). The Seed-based Mapping (SDM) method is a statistical approach for examining neural activity or structure variations. The procedure comprised: (1) extracting peak coordinates and t-values from differential brain regions, with coordinates uniformly converted to MNI format using the SDM website’s conversion function; (2) rebuilding differential brain region maps in the software, adjusting voxel values for alignment with the original study outcomes (for studies providing multiple coordinates, SDM aggregated the results); and (3) determining effect values and variances for the brain maps based on t-statistics (or value of ps or z-scores). Following this, a meta-analysis was conducted on the standardized brain maps in accordance with set criteria. Analysis was conducted in MNI spatial coordinates with statistical thresholds established at *p* < 0.005, Z > 1, and cluster size >10, equivalent to a corrected *p* < 0.05. Anatomical visuals were then generated from the meta-analysis findings using MRIcron software.

Based on the study design, the results were categorized into two subgroups: resting-state fMRI studies that utilized true and sham acupoint controls, and task-state fMRI studies where the needle was retained without initial stimulation and subsequently compared with a stimulated condition. We conducted subgroup analyses on both datasets. To consider the potential impacts of needle duration, depth, and diameter on the outcomes, meta-regression was implemented to assess the associations between these variables and alterations in specific brain regions. A more stringent threshold test (*p* < 0.0005) was adopted to reduce the likelihood of spurious results. Furthermore, in analyzing the meta-analysis outcomes at the functional network tier, the LR3-activated regions were mapped to 14 established functional networks, including the Anterior salience network, Auditory network, Basal ganglia network, Dorsal default mode network (dDMN), High visual network, Language network, Left executive control network (LECN), Posterior salience network, Precuneus network, Primary visual network, Right executive control network (RECN), Sensorimotor network, Ventral default mode network (vDMN), and Visuospatial network.

### 2.5 Stability analysis

To ensure the reliability of our findings, a Jackknife sensitivity analysis was conducted using SDM software. This analysis systematically omits one study at a time to determine the consistency of the results. Concurrently, the software was employed to assess inter-study heterogeneity, with criteria of *p* > 0.1 and I2 < 50% indicating homogeneity among the combined effect sizes. Additionally, potential publication bias was evaluated using Egger’s test, where a value of p less than 0.05 indicates significant bias.

## 3 Result

### 3.1 Study characteristics

**Figure 1:**
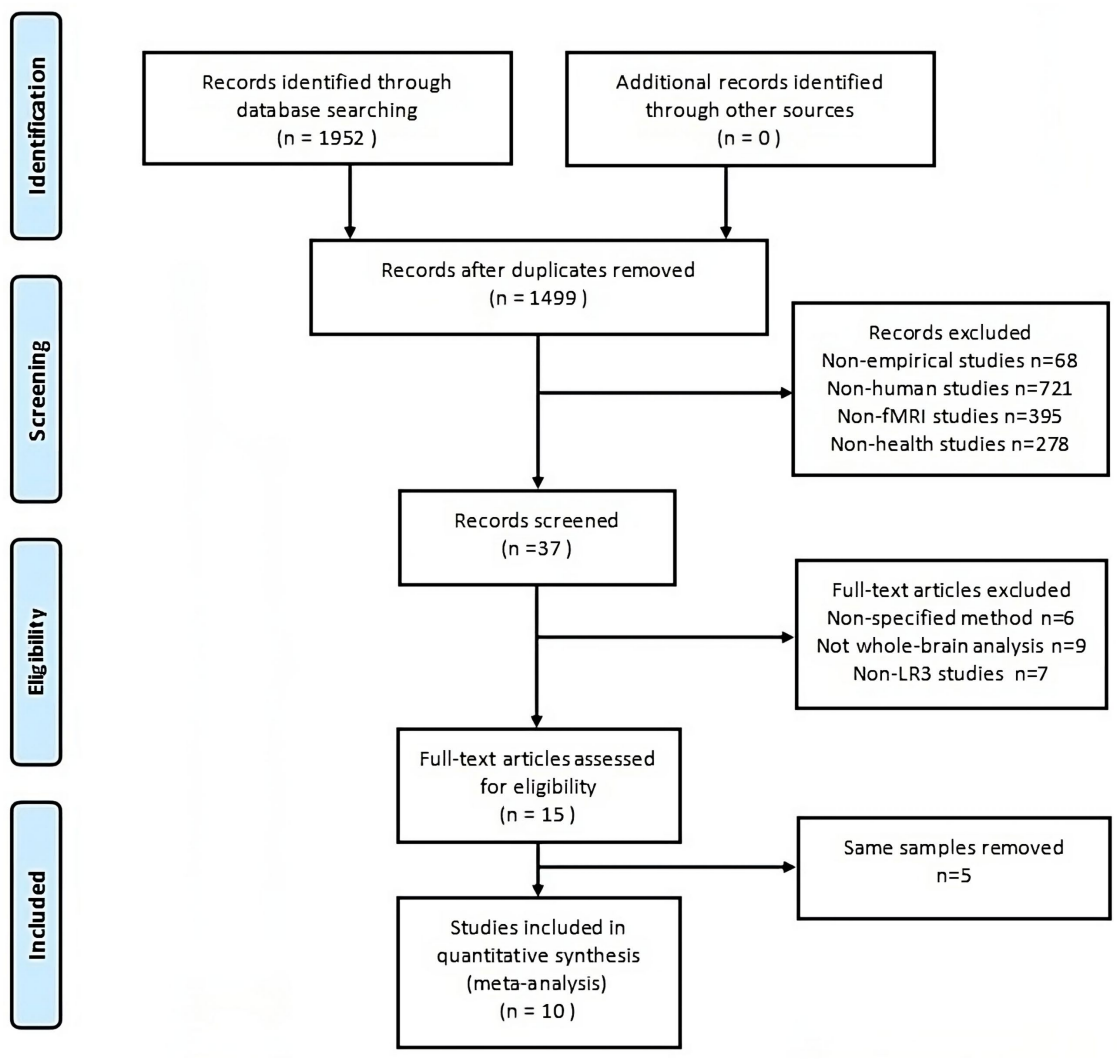
The PRISMA flow diagram illustrates comprehensive search and selection procedures. fMRI, functional magnetic resonance imaging.

The process of screening and selecting articles is detailed in Figure 1. From the combined search and screening efforts, 1,952 publications emerged. Ten articles (Liu H. et al., 2006; Wei et al., 2006; Li et al., 2008; Fang et al., 2009; Claunch et al., 2012; Wang et al., 2013; Zheng et al., 2016; Chen et al., 2019; Xiaoling et al., 2020) were ultimately included in the meta-analysis, as delineated in Table 1. No further pertinent literature emerged from the references of the selected articles. The studies chosen comprised 163 healthy subjects and 156 control subjects. Half of these studies contrasted actual acupuncture points with sham points, and MRI scans were performed post-acupuncture. Conversely, the remaining five studies employed a task-state fMRI design wherein the stimulation task was executed during scanning. Here, the control group symbolized a baseline state with acupuncture needles retained without stimulation. Both methodologies are prevalently adopted to discern the immediate acupuncture effects.

**Table 1:** The characteristics of the included studies.

Study	MRI scanner	Processing software	Coordina system	Activation type	Subjects, n		Mean age(SD)	Statistical threshold	Time	Quality scores
					Patients	Controls				
Murong (2006)	1.5 T,Siemen,Sonata	SPM	Tal	MA vs. Sham	12	11	26.8(3.6)	*p* < 0.001, uncorrected	180S	20
zheng et al. (2016)	3.0 T,GE	SPM	MNI	MA vs. Sham	15	15	21.87(1.18)	*p* < 0.05, corrected	60s	17
li et al. (2008)	1.5 T,Siemen,Sonata	SPM	Tal	MA vs. Sham	10	7	23.7(3.6)	*p* < 0.001, uncorrected	180S	18
Liu et al. (2006)	1.5 T,Siemen,Sonata	SPM	Tal	MA vs. Sham	9	8	26.8(3.6)	*p* < =0.01, uncorrected	180S	18
Fang et al. (2009)	1.9 T,GE	SPM	MNI	Baseline-activation	6	/	25(3)	*p* < 0.001, uncorrected	60s	18
chen et al. (2019)	1.5 T,Signa	SPM	MNI	Baseline-activation	10	/	22(3)	*p* < 0.005, uncorrected	30s	18
Xiaoling et al. (2020)	3.0 T,Philips	SPM	MNI	Baseline-activation	15	/	25.36(1.72)	*p* < =0.001, uncorrected	60s	19
Wei et al. (2006)	1.5 T,Siemen,Sonata	SPM	Tal	MA vs. Sham	10	8	26.5(3.4)	*p* < 0.001, uncorrected	180S	20
Wang et al. (2013)	1.5 T,Siemen,Sonata	AFNI	Tal	Baseline-activation	30	/	28.6(8.05)	*p* < 0.005, corrected	120S	19
Claunch et al. (2012)	1.5 T,Siemen,Sonata	AFNI	Tal	Baseline-activation	46	/	29.1(7.63)	*p* < 0.05, corrected	120S	20

MA, manual acupuncture.

### 3.2 Main SDM analysis

**Figure 2:**
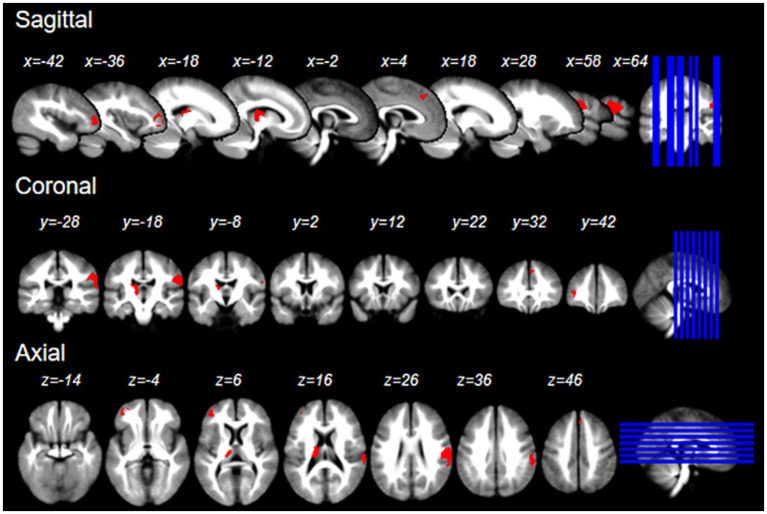
The brain regions activated by acupuncture at LR3 in the x, y, and z Planes.

We refined the primary SDM analysis outcomes by setting a threshold at *p* < 0.005. This identified 8 brain regions with positive activation and 16 with negative activation. To enhance the robustness of our findings, we excluded regions demonstrating significant heterogeneity (I2 > 50) or substantial bias (Egger *p* < 0.05). These results were then cross-verified using the Jackknife sensitivity analysis, retaining only those brain regions that consistently appeared in more than half of the ten analyses. It is noteworthy that no brain regions exhibited negative activation. Consequently, we pinpointed four brain regions with consistent positive activation: the Right postcentral gyrus, Left thalamus, Left middle frontal gyrus, and Right superior frontal gyrus (refer to Table 2 and Figure 2).

**Table 2:** The brain regions activated by acupuncture at LR3.

Anatomical region	MNI Coordinate	SDM-Z	*p-*value	Voxels	Heterogeneity	Sensitivity analyses	Egger test (*p-*value)
Right postcentral gyrus	58,-18,30	3.021	<0.01	711	No	10 out 10	0.446
Left thalamus	-16,-22,16	2.467	<0.01	234	No	10 out 10	0.271
Left middle frontal gyrus	-42,48,2	2.49	<0.01	183	No	10 out 10	0.468
Right superior frontal gyrus	4,30,42	2.086	<0.01	98	No	6 out 10	0.606

### 3.3 Subgroup analysis

**Figure 3:**
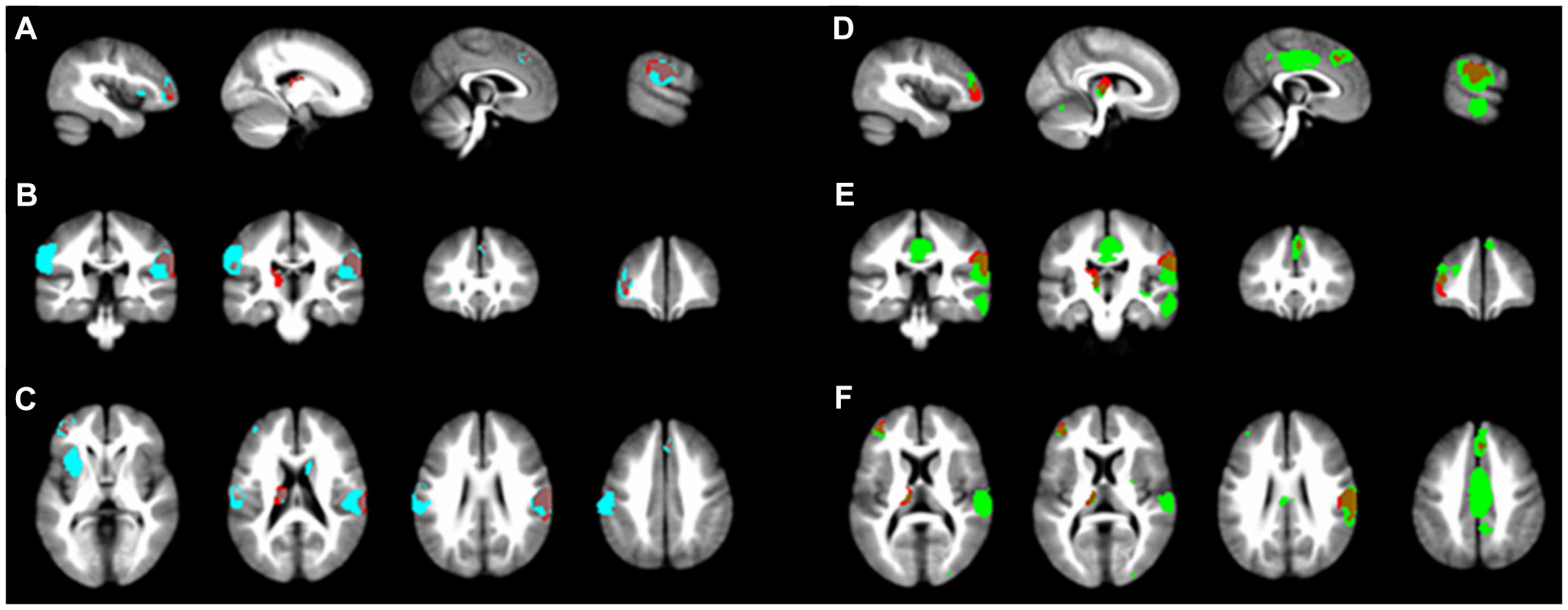
Subgroup analysis results. The main SDM analysis findings are marked in red. **(A-C)** illustrate the activated brain regions for the ts-fMRI subgroup across the x, y, and z planes, respectively. In contrast, **(D-F)** depict the activated regions for the rs-fMRI subgroup on the matching x, y, and z planes.

Two subgroup analyses were performed: one for the five studies using rs-fMRI and the other for the five studies using ts-fMRI. The findings from both subgroups largely mirrored the primary SDM analysis results (refer to Figure 3). Notably, the activated brain regions identified in the ts-fMRI subgroup aligned more with the main SDM findings, showing complete overlap in all four regions. In contrast, the rs-fMRI subgroup identified only three regions: Left middle frontal gyrus, Left thalamus, and Right postcentral gyrus. Additionally, the ts-fMRI subgroup exhibited fewer brain regions with unrelated activation than the rs-fMRI subgroup.

### 3.4 Meta-regression analysis

A regression analysis assessed the influence of needle retention duration and needling depth on brain function. The meta-regression data (refer to Table 3) indicate a statistically significant positive association between the activation of the right superior frontal gyrus and needle retention time (*p* < 0.00005). This implies that prolonged needle retention time correlates with increased activation of this region. Conversely, the needling depth did not significantly impact the functionality of this brain region.

**Table 3:** The brain region associated with the duration of needle retention.

MNI coordinate	SDM-Z	*p*	Voxels	Description
54,-24,12	3.854	0.00003612	127	Right superior frontal gyrus

### 3.5 Mapping of brain functional networks

**Figure 4:**
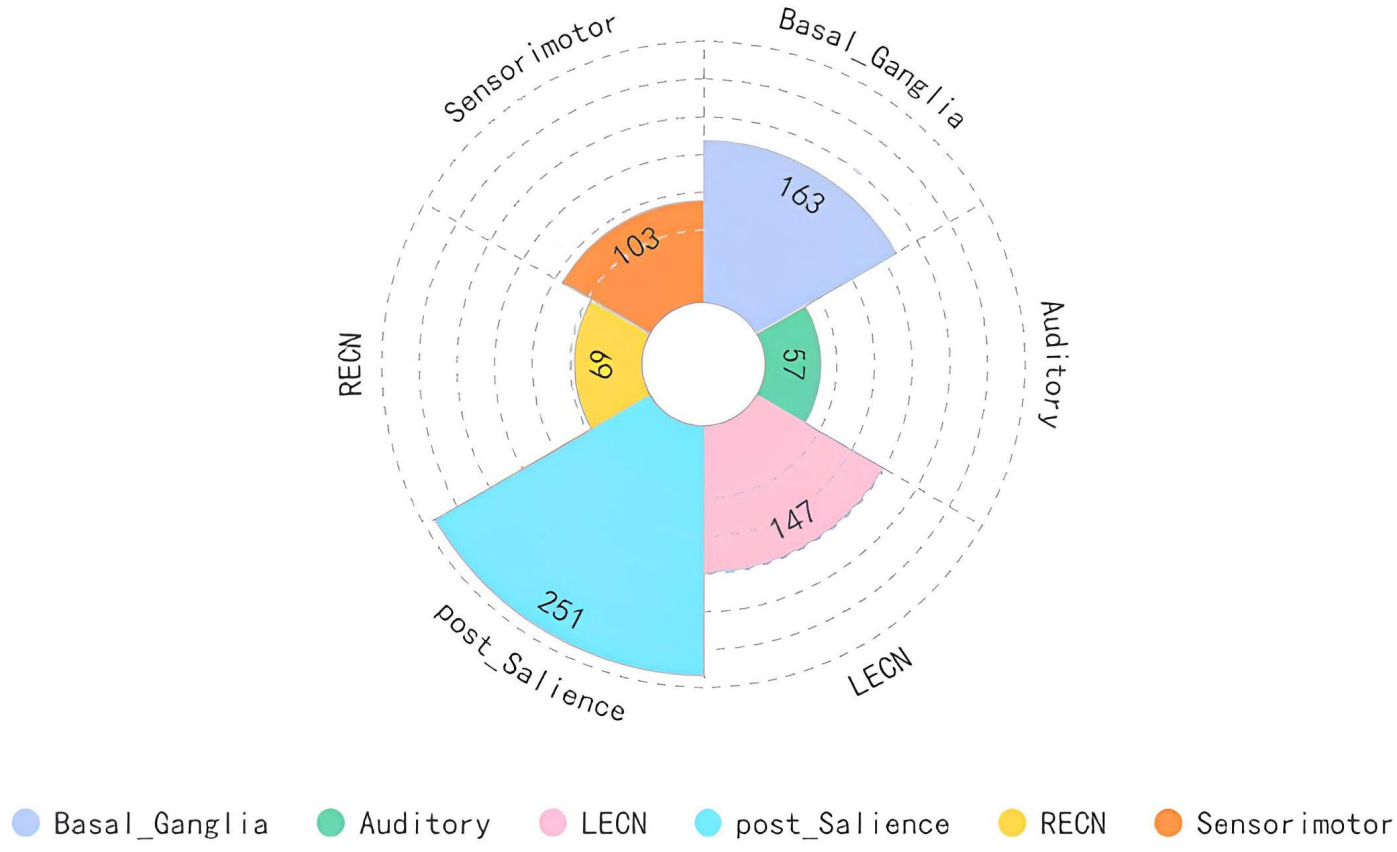
Main analysis results mapped to brain functional network. LECN, left executive control network; RECN, right executive control network.

We mapped the brain regions activated by acupuncture at the LR3 to 14 classical brain functional networks. The findings, detailed in Figure 4 and Table 4, demonstrate that the overlap between the activated regions and 6 functional networks contains more than 50 voxels. Predominantly, these voxel mappings are distributed across the Basal ganglia network, Auditory network, LECN, Posterior salience network, RECN, and Sensorimotor networks.

**Table 4:** Functional network mapping of the brain regions activated by acupuncture at LR3.

Network	Overlap voxels
Anterior salience network	91
Auditory network	57
Basal ganglia network	163
Dorsal default mode network	0
High visual network	0
Language network	0
Left executive control network	147
Posterior salience network	251
Precuneus network	0
Primary visual network	0
Right executive control network	69
Sensorimotor network	103
Ventral default mode network	0
Visuospatial network	5

## 4 Discussion

To our knowledge, this study represents the first application of the SDM technique to examine how acupuncture at the LR3 acupoint modulates healthy subjects’ brain activation patterns. Our meta-analysis included 10 coordinate-based fMRI studies. The pooled results demonstrate positive activation in the right postcentral gyrus, left thalamus, left middle frontal gyrus, and right superior frontal gyrus following acupuncture stimulation at the LR3 acupoint, with no regions exhibiting negative activation. Further subgroup analysis shows greater concordance of activated brain regions with the main SDM analysis findings in the ts-fMRI subgroup. Regression analysis also indicates a significant positive correlation between the intensity of activation in the right superior frontal gyrus and needle retention time. Through functional network mapping, we identify that brain regions activated by LR3 acupuncture predominantly align with functional networks, including the Basal ganglia network, Auditory network, LECN, Posterior salience network, RECN, and Sensorimotor networks.

LR3, situated on the Liver Meridian of Foot-Jueyin, is designated as the ‘Yuan-Primary point’ of this meridian and has traditionally been a pivotal point for addressing liver and gallbladder-related ailments. TCM posits that this acupoint serves various functions, such as regulating liver activity, enhancing qi and blood circulation, and alleviating pain. In contemporary clinical practice, the LR3 is extensively employed to manage various conditions, including hypertension, pain, depression, hepatitis, and cognitive deficits. Several animal studies (Lu J. et al., 2017; Yang et al., 2017; Luo et al., 2019; Li et al., 2023) have demonstrated that acupuncture at the LR3 can markedly reduce blood pressure in spontaneously hypertensive rats, with varying acupuncture techniques eliciting differential hypotensive effects. Additionally, the acupuncture at LR3 can prevent hippocampal neuronal apoptosis by augmenting the Bcl-2/Bax ratio in the hippocampal region, thereby reducing brain damage induced by hypertension (Lu J. et al., 2017). Correspondingly, in clinical trials involving Alzheimer’s disease (AD) patients, favorable outcomes were observed following acupuncture treatment at the LR3 (Liang et al., 2014; Ji et al., 2021).

In the present study, the right posterior central gyrus was identified as the region with the most significant activation. LR3 is located on the dorsal aspect of the foot, specifically in the anterior depression where the first and second metatarsal bones converge. At this site, somatosensory nerve impulses ascend via the peroneal nerve, arrive at the lumbar plexus of the spinal nerves, penetrate the spinal cord, and undergo synaptic transmission in the posterior horn of the spinal cord gray matter. Subsequently, these impulses ascend the spinothalamic tract, culminate in the anteroposterior nucleus of the contralateral thalamus, undergo another round of synaptic transmission, then ascend the internal capsule to the cerebral cortex, eventually arriving at the posterior central gyrus of the parietal lobe. The posterior central gyrus is part of the primary somatosensory cortex (S1), a critical region for receiving external stimuli and generating sensations, tasked with processing tactile, thermal, proprioceptive, and pain sensations from both internal and external sources (Ngo et al., 2021). The S1 region is instrumental in encoding various sensory characteristics of pain (Vierck et al., 2013), encompassing the perception and modulation of painful and non-painful somatic sensations (Bushnell et al., 1999). During acute muscle pain, the S1 region of the hemisphere corresponding to the side-inducing pain is activated, diminishing the excitability of S1 to lessen the processing of non-painful input information (Burns et al., 2016). In patients with a toothache, activation of the bilateral S1 has been noted. The higher the pain score, the weaker the connection between the bilateral S1 (Hu et al., 2018). Moreover, chronic pain is invariably linked with functional reorganization of S1 (Gustin et al., 2012). The plasticity of the S1 cortex may contribute to chronic pain (Kim et al., 2017). Shi et al. (2020) discovered that the S1 brain region of rats experiencing pain was activated. Electroacupuncture can mitigate pain perception and ameliorate pain-related emotions by modulating the neural oscillatory activity of S1, thus alleviating chronic pain.

Recent neurofunctional studies have shown that in addition to the primary somatosensory cortex (S1), the thalamus is one of the most frequently activated brain regions in pain response (Kaplan et al., 2019). The thalamus, a complex subcortical structure, contains specific nuclei that receive inputs from the cortex, subcortex, and cerebellum and project to various cortical functional areas (Farbota et al., 2012). Importantly, since most connections between cortical and subcortical regions primarily involve the thalamus, it is central to many brain functions and is a pivotal node in sustaining brain function stability (Farbota et al., 2012; Kumar et al., 2017). Specific sensory nuclei within the thalamus integrate stimuli from the body and its surface. In pain perception, the lateral thalamocortical pathway predominantly encodes sensory discrimination, whereas the medial thalamocortical pathway primarily encodes the affective aspect of pain (Groh et al., 2018). Evidence suggests that exposure to acute noxious stimuli markedly enhances thalamic activation (Kobayashi et al., 2009; Friebel et al., 2011). Furthermore, the thalamus is vital in managing both acute and chronic pain (Lenz et al., 2004). Some studies indicate that acupuncture at LR3 deactivates the limbic-paralimbic-neocortical network, transitioning pain-activated brain regions into a suppressed state, thereby generating an analgesic effect (Hui et al., 2000, 2009). In contrast, other research proposes thalamic activation following LR3 acupuncture (Wu et al., 2010; Liu et al., 2013). It is worth noting that most studies are conducted on healthy individuals, necessitating further research on patients with pain. Considering acupuncture’s bidirectional regulatory properties, it might have distinct effects in pathological versus physiological conditions. Future studies on pain patients could provide insights into the mechanisms through which LR3 mediates its analgesic effects via thalamic regulation.

Additionally, the superior frontal gyrus is closely related to the perception of stress. When it comes to mental disorders associated with abnormal stress perception, such as post-traumatic stress disorder, major depressive disorder, generalized anxiety disorder, and substance abuse, the spontaneous activity of the superior frontal gyrus shows an increasing trend. Several fMRI studies (Chen et al., 2018; Ma et al., 2019) also found that the heightened spontaneous activity of the superior frontal gyrus is directly linked to reduced emotional regulation capability, decreased life satisfaction, and pessimistic personality traits. These emotional and cognitive changes further influence the individual’s perception of stress. Cognitive inhibition, which involves controlling one’s thoughts to maintain emotional stability, has been found to have a positive correlation with the gray matter volume of the right superior frontal gyrus (Lu et al., 2022). Additionally, some studies indicated that the weakened functional connection between the right middle temporal gyrus and the right superior frontal gyrus is associated with procrastination, further confirming the pivotal role of the right superior frontal gyrus in emotional regulation (Zhang Y. et al., 2022). This result is consistent with findings from several previous studies, which indicate that during acupuncture at the LR3, brain regions primarily governing emotional regulation demonstrate specific activation patterns (Wu et al., 2014; Zhang et al., 2015). LR3 is the yuan-primary point of the Liver Meridian of Foot-Jueyin. According to Traditional Chinese Medicine (TCM) theory, liver regulation can affect emotional fluctuations in individuals. Previous literature has also noted that the LR3 is frequently used in treating emotional disorders such as depression and anxiety (Wu and Liu, 2008; Wong et al., 2021). Therefore, we hypothesize that the right superior frontal gyrus may be the central pathways through which LR3 influences emotion generation, processing, and regulation.

The middle frontal gyrus (MFG) plays a central role in language functions, encompassing not only speech and semantic processing but also verb processing. When reading Chinese characters, the left MFG demonstrates notably heightened activation, displaying a marked differential in activation compared to reading English or other Latin scripts. Notably, reduced activation of the left MFG is identified as a primary pathological characteristic of developmental reading disorders in Chinese (Wen et al., 2017; Li et al., 2022). While a consensus regarding the role of this brain region in reading Chinese characters remains elusive, the prevailing perspective associates it with visual–spatial analysis (Liu C. L. et al., 2006). Some research posits that the left MFG is intricately connected to general working memory and contributes to the processing of visual attributes (Kurosaki et al., 2016). Alternatively, evidence suggests the MFG is pivotal in assimilating spatial information, necessitating intricate spatial frequency processing, especially when discerning the component relationship of Chinese characters (Liu C. Y. et al., 2022). Although limited studies have examined the association between acupuncture at the LR3 and language, it remains uncertain whether a direct link exists between MTG activation and the treatment of aphasia and related disorders through acupuncture at LR3. Further research is imperative to elucidate this relationship.

In current randomized controlled trials (RCTs) evaluating acupuncture, sham acupuncture is commonly utilized as a standard control method. An optimal acupuncture control strategy should exhibit no notable difference from authentic acupuncture in both visual and tactile senses and should refrain from inducing specific therapeutic effects (Streitberger and Kleinhenz, 1998). A multitude of acupuncture research employs diverse sham acupuncture strategies, which scholars have assessed thoroughly. Prevalent methods encompass superficial needling outside of meridians and acupuncture points, non-insertion outside of meridians and acupuncture points, and non-insertion at acupuncture points. Nonetheless, these strategies frequently need to fulfill the ideal requisites for acupuncture controls (Liu et al., 2023). In fMRI studies, the use of sham points may influence peripheral nerves, thereby modifying the functional activity of specific brain regions (Tsukayama et al., 2006). Task-state fMRI maintains the needle at specific points, contrasting resting and stimulation states to mitigate interference with unrelated nerves. These two strategies, to this point, have yet to undergo extensive comparative analysis. We performed a subgroup analysis aiming to discern which strategy proves more effective in fMRI studies. In alignment with our preliminary expectations, the results of the task-state control strategy demonstrated greater consistency with the primary outcomes, concurrently exhibiting less activation in unrelated brain regions. In contrast, the sham point strategy revealed inferior consistency and potentially incited activation in additional unrelated brain regions. This intimates that the task-state strategy can more accurately reflect the alterations in brain function induced by acupuncture treatment. However, given the constrained sample size, our conclusions necessitate further evidential support.

According to the meta-analysis, there was no significant correlation found between the depth of acupuncture and the diameter of the needle. This observation is in line with the fundamental principles of acupuncture in Traditional Chinese Medicine, which suggests that “deqi” is an indication of successful treatment. Therefore, subtle variations in needle diameter and depth are considered to have a minimal impact on the effectiveness of therapy. Upon further analysis, it was observed that as the duration of acupuncture treatment increased, the functional intensity of the right superior frontal gyrus also amplified. This area is crucial in regulating emotions and cognitive functions, highlighting the potential advantages of extending acupuncture treatment for depression and other related mental conditions. Additionally, a supporting meta-analysis reinforces the idea that extended treatment duration augments therapeutic efficacy in depression (Yang et al., 2022; Tu et al., 2023). Future studies should aim to ascertain the ideal duration for acupuncture treatment at the LR3 for mental disorder interventions.

In the primary analysis, four distinct brain regions were identified and classified into several networks, namely Basal ganglia network, Auditory network, LECN, Posterior salience network, RECN, and Sensorimotor networks. Research on depression-associated brain networks highlights the significance of the DMN, CEN, and SN, emphasizing their central roles in depression progression (Uddin et al., 2011). The Default Mode Network (DMN) is linked with self-awareness, autobiographical memory, and social cognition, showing heightened activity during resting states (Fox et al., 2005). The Central Executive Network (CEN) contributes to cognitive functions, decision-making, and working memory and is integral to goal-directed tasks and emotional regulation (Fox and Raichle, 2007; Brakowski et al., 2017). The Salience Network (SN) serves as a bridge between the DMN and CEN, orchestrating the integration of internal and external stimuli, and is pivotal in coordinating interactions among DMN, CEN, and SN (Seeley et al., 2007). The Basal Ganglia are instrumental in information relay and modulation within the brain, establishing intricate ties with the cerebral cortex and influencing motor control, emotional processing, and other associative functions (Calabresi et al., 2014). Moreover, brain network mapping research indicates that acupuncture at the LR3 can modulate emotions, alter bodily perceptions and movements, and bolster learning and memory capabilities in patients.

Several limitations in this study need to be considered. Firstly, the number of included studies is relatively limited, and the sample sizes within each study are also modest. Secondly, some studies employed unilateral interventions at the LR3, which might introduce disparities in functional effects between hemispheres, potentially influencing the accuracy of the results. Although some research indicates minimal differences between unilateral and bilateral acupuncture effects, further validation remains essential. Moreover, the publication years of the included studies span a broad range, and discrepancies in experimental designs and fMRI data processing methods are evident. These factors necessitate a prudent interpretation of the results. This study is intended to guide subsequent research in this field. We advocate for more extensive studies in the future to validate and enrich the findings presented here.

## 5 Conclusion

Acupuncture at LR3 in healthy individuals mainly activates the right postcentral gyrus, left thalamus, left middle frontal gyrus, and right superior frontal gyrus. Ts-fMRI may provide a more precise depiction of the brain regions’ responses to stimulations by LR3. An extended needle retention duration may enhance the activation intensity within the right superior frontal gyrus. Moreover, LR3 acupuncture affects multiple brain functional networks. Our results offer neurological support for acupuncture therapy and enhance insight into its underlying mechanisms.

## Data availability statement

The original contributions presented in the study are included in the article/supplementary material, further inquiries can be directed to the corresponding author.

## Author contributions

YR: Conceptualization, Project administration, Writing – original draft. LG: Methodology, Writing – review & editing. JW: Data curation, Writing – review & editing.
